# Promotions of breastmilk substitutes, commercial complementary foods and commercial snack products commonly fed to young children are frequently found in points‐of‐sale in Bandung City, Indonesia

**DOI:** 10.1111/mcn.12808

**Published:** 2019-06-21

**Authors:** Dian N. Hadihardjono, Mackenzie Green, Ame Stormer, Agustino, Doddy Izwardy, Mary Champeny

**Affiliations:** ^1^ Helen Keller International New York New York USA; ^2^ Direktorat Gizi Masyarakat‐Kementerian Kesehatan RI Jakarta Indonesia

**Keywords:** breastmilk substitute, commercially produced complementary food, commercially produced snack food, International Code of Marketing Breast‐milk Substitutes, point‐of‐sale, promotion

## Abstract

Few studies have documented the marketing of commercial foods and beverages for infants and young children in West Java, Indonesia. To assess the prevalence of promotions at points‐of‐sale for commercially produced products commonly fed to young children in Bandung City, 43 small and large stores were visited in 2017. Promotions for breastmilk substitutes (BMS), commercially produced complementary foods (CPCF), and select types of commercial snack products were photographed and information recorded on promotion characteristics. There were 402 and 206 promotions observed with BMS and CPCF products, respectively. Sixteen promotions with BMS products for infants under 12 months were found in 42.9% of stores selling BMS, violating national regulations. Almost all BMS promotions (98.3%) included BMS products for ages 1 year and above (“growing‐up milks”). Of all BMS products available for sale, half of all infant/follow‐up formula and 77.2% of growing‐up milks were promoted. CPCF were found in 97.7% of stores, and 81.0% of these stores had promotions; 70.5% of all available CPCF products were promoted. Of the 2,451 promotions observed for commercial snack products, 17.3% used promotional techniques targeting young children or caregivers. Joint‐promotions were common, with BMS and CPCF marketed in combination with commercial snack products; 49.0% of BMS promotions were joint BMS‐snack promotions, and 80.0% or more of infant/follow‐up formula promotions included a commercial snack. Revising and enforcing infant food and beverage marketing regulations to ensure consistency with global standards are necessary to protect and promote optimal infant and young child feeding in Indonesia.

**Key messages**

- Promotions for commercial infant and young child feeding products were wide‐spread in points‐of‐sale in Bandung City, Indonesia.
- Violations were observed of Indonesian regulations and the International Code of Marketing of Breast‐milk Substitutes.
- Almost all BMS promotions included a growing‐up milk and 49.0% included a joint‐promotion with commercial snack products.
- Nearly one‐fifth of promotions for five categories of commercial snack products were targeted to children or caregivers.
- Enforcement of national regulations is necessary to prevent promotions that can negatively impact child feeding. Additional regulations should be considered to restrict promotion of BMS for older children, joint‐promotion of BMS with commercial snacks, inappropriate promotion of commercial complementary foods and child‐targeted advertising of unhealthy commercial snack products.

## 1 INTRODUCTION

The World Health Organization (WHO) and United Nations International Children's Emergency Fund (UNICEF) recommendations for optimal infant and young child feeding include exclusive breastfeeding for the first 6 months of life, at which point safe and appropriate complementary foods should be gradually introduced to a child's diet alongside continued breastfeeding up to 2 years and beyond (World Health Organization & United Nations International Children's Emergency Fund [WHO & UNICEF], 2003). Research throughout the world has documented the health and nutritional benefits of breastfeeding (Victora et al., 2016) and consumption of nutrient‐rich and appropriate complementary foods during a critical period of growth and development (Shrimpton et al., 2001; WHO & UNICEF, 2003).

Infant and young child feeding practices in Indonesia do not meet WHO and UNICEF recommendations for optimal nutrition and development (Beal, Tumilowicz, Sutrisna, Izwardy, & Neufeld, 2018; Ng, Dibley, & Agho, 2012). Only 41.5% of children nationwide are exclusively breastfed for the first 6 months of life, and just over half of children (55.3%) are still breastfeeding at 20–23 months (Badan Pusat Statistik [BPS], National Population and Family Planning Board [BKKBN], Kementerian Kesehatan [Kemenkes], & ICF International, 2013). The use of breastmilk substitutes (BMS) is highly prevalent, with over one‐third of breastfed infants under 6 months fed BMS (BPS, BKKBN, Kemenkes, & ICF International, 2013). Complementary foods are often introduced too early (BPS, BKKBN, Kemenkes, & ICF International, 2013; Muslimatun & Wiradnyani, 2016), and a number of studies have documented micronutrient deficiencies among children in the complementary feeding period (Diana et al., 2017; Fahmida & Santika, 2016; Muslimatun & Wiradnyani, 2016; Santika, Februhartanty, & Ariawan, 2016). There is also growing evidence that commercial snack foods, with high levels of salt, sugar and fat, are becoming increasingly common in diets of infants and young Indonesian children (Green et al., 2019; Imanningsih, Jahari, Permaesih, Chan, & Amarra, 2018; Purwestri et al., 2018; Sekiyama, Roosita, & Ohtsuka, 2012; White et al., 2016), potentially displacing breastmilk and other nutrient‐rich foods (Pries et al., 2017). A study in Central Java found that stunted children 6–59 months had significantly higher snack consumption than non‐stunted children in the last 24 hr (Purwestri et al., 2018).

Inappropriate promotion of food products can negatively influence feeding practices and diets of infants and young children (Piwoz & Huffman, 2015; Rollins et al., 2016; WHO, 2017), and WHO has released guidelines to regulate the marketing activities of manufacturers. The *International Code of Marketing of Breast‐milk Substitutes* (the Code; WHO, 1981) prohibits all advertising to the public of BMS, including at points‐of‐sale, and World Health Assembly Resolution (WHA) 69.9 and the WHO *Guidance on Ending the Inappropriate Promotion of Foods for Infants and Young Children* affirm that commercial milk products for children up to 36 months of age are BMS (WHO, 2016). The Code has been partially adopted in national regulations in Indonesia, encompassed by Health Law 36/2009 (Badan Pembinaan Hukum Nasional, 2009), Government Regulation 33/2012 on Exclusive Breastfeeding (Ministry of Health [MOH], 2012), and the Food Label and Advertisement Regulation 69/1999 (Badan Pengawas Obat Dan Makanan, 1999). These regulations are narrower in scope than the Code, only prohibiting the promotion of BMS products for infants up to 1 year of age (MOH, 2012), and they lack provisions on contact with mothers, use of pictures and text idealizing BMS and recommendations on BMS labels (International Baby Food Action Network, 2015; WHO & UNICEF, 2018).

WHA 69.9 and the WHO *Guidance on Ending the Inappropriate Promotion of Foods for Infants and Young Children* also govern the marketing of commercially produced complementary foods (CPCF), stating that CPCF may be promoted if they meet relevant guidelines for “composition, safety, quality and nutrient levels” (WHO, 2016). CPCF must not be promoted or otherwise represented as appropriate for feeding children under 6 months and must not discourage breastfeeding; cross promotion of BMS and CPCF products is prohibited. Indonesian policy only restricts mass media advertisement of food products for infants under 12 months (MOH, 2012), with no regulation on the promotion of CPCF products at retail locations, including cross promotion with BMS, which has been documented in Indonesia (Durako & Lo, 2016).

The 2010 WHO *Set of Recommendations on the Marketing of Foods and Non‐alcoholic Beverages to Children* encourages countries to enact policies which reduce children's exposure, directly and indirectly, to marketing of foods with high saturated fat, trans‐fatty acids, free sugars or salt (WHO, 2010). Inclusion of nutrient‐poor snack foods and beverages in regulations controlling inappropriate promotions of foods for young children is increasingly important as consumption becomes more prevalent (Pries, Filteau, & Ferguson, 2019) and habits and taste preferences established in childhood have been linked to long‐term eating preferences (Beauchamp & Mennella, 2009; Ventura & Mennella, 2011). Indonesia lacks such regulation, in spite of the growing availability of commercially produced snack foods (Baker & Friel, 2016; Shrimpton & Rokx, 2013). An U.S. Department of Agriculture report noted $7.1 billion in sales of packaged commercial snacks in Indonesia in 2016 and that growth of the food processing industry was due in part to “aggressive promotional activities” (U.S. Department of Agriculture Foreign Agricultural Service, 2018).

Violations of the Code and national law at points‐of‐sale are reported in Indonesia (Durako & Lo, 2016; Hidayana, Februhartanty, & Parady, 2017; International Baby Food Action Network, 2014); however, there is little evidence on the prevalence of in‐store promotions for CPCF or commercially produced snack products, their joint‐promotion with BMS and whether commercial snack promotions in Indonesia target caregivers or young children. This study was conducted to document the commercial marketing that caregivers are exposed to at retail locations in Bandung City. The capital of West Java, Indonesia's most populous province, Bandung City has high under‐five stunting (32.2%; MOH, 2013) and only two‐thirds of children 6–23 months of age achieve a minimum acceptable diet according to WHO guidelines (Santika et al., 2016; WHO, 2010). Recent research by Green et al. (2019) in Bandung City found prevalent consumption of BMS (49.5%), CPCF (37.4%), commercial snack foods (81.6%) and sugar‐sweetened beverages (40.0%) among children 6–35 months of age. Caregivers interviewed in the study reported near universal exposure to commercial promotions for these products (93.3% BMS, 97.0% CPCF, 97.5% commercial snack/beverage; Green et al., 2019 & Helen Keller International [HKI] & MOH, 2018a, 2018b). Understanding the extent of commercial marketing in retail locations is needed for the development and strengthening of national regulations on inappropriate promotion to help protect infant and young child feeding practices in Indonesia.

## 2 METHODS

This cross‐sectional study documented availability and promotional practices in retail locations for BMS and CPCF and assessed promotions for a subset of commercial snack products commonly fed to children under 3 years of age in Bandung City.

### 2.1 Sampling of points‐of‐sale

Sampling of points‐of‐sale for this assessment was based on the Network for Global Monitoring and Support for Implementation of the International Code of Marketing of Breast‐milk Substitutes and Subsequent Relevant World Health Assembly Resolutions (NetCode) Protocol for Periodic Assessments (WHO & UNICEF, 2017). A total of 43 points‐of‐sale were sampled: 10 large stores and 33 small stores, all carrying at least one BMS or CPCF product. Large stores were purposively sampled in consultation with local officials and non‐governmental organizations working on child health and included four grocery stores/supermarkets, four hypermarkets and two baby stores. Seven of the locations were national or local chain stores. The 10 locations were chosen for their wide variety and volume of products that would be representative of availability in Bandung City.

For this study, small stores could include corner stores (*warung*/*kiosks*), neighbourhood cooperative grocery stores (*koperasi*), minimarts and pharmacies (*apotiks*). Small stores were sampled for their proximity to public sector health facilities offering child health services (WHO & UNICEF, 2017). The 33 facilities were identified in coordination with the Bandung City Health Office in preparation for a survey with mothers of young children (Green et al., 2019). Using Google Maps and Google Street View, four small stores in closest proximity to each health facility were listed. During data collection, stores were visited in order of proximity and the first found to sell a BMS or CPCF product was included for that facility. If a store not identified through Google Maps and Street View was found in closer proximity and met study criteria, it was used instead. In total, 19 *warung/kios*, 12 minimarts, 1 *koperasi* and 1 a*potik* were surveyed; 11 minimarts and the 1 *apotik* were national chains.

### 2.2 Product definitions

This study focused on three types of commercial products: BMS, CPCF and select categories of commercially produced snacks commonly fed to young children. BMS products were defined as any formula, milk or milk‐like product, in either liquid or powdered form, marketed for feeding infants and young children under 3 years of age (WHO, 2016). BMS products were sub‐categorized as:

1. infant formula for infants 0–5 months of age;
2. follow‐up formula for 6–11 months; and
3. growing‐up milks for 12–35 months.

CPCF were foods marketed as suitable for feeding young children if they met at least one of the following criteria: (1) recommended for introduction at an age of less than 3 years; (2) labelled with the words “baby”, “toddler,” “young child,” or synonym; or (3) in any other way were presented as being suitable for children under the age of 3 years (WHO & UNICEF, 2017). CPCF were sub‐categorized into:

1. infant cereal, including instant *bubur* (rice porridge);
2. grain snacks/finger foods, including products such as biscuits/cookies, puffs or rusks;
3. pureed foods and infant meals, which may include cereal, pasta, meat, poultry, fish, eggs, fruits and/or vegetables;
4. infant pudding (instant milk/gelatine pudding); and
5. other CPCF products, including tea, juice, olive oil or *abon* (finely shredded meat).

Commercial snacks were defined as manufactured, packaged products marketed for consumption by all ages (Pries et al., 2016). Sub‐categories of commercial snacks commonly fed to young children were identified through previous literature (Fahmida & Santika, 2016; GAIN & MOH, 2013; Muslimatun & Wiradnyani, 2016; Pries et al., 2017; Sekiyama et al., 2012; White et al., 2016) and discussions with local experts. Sub‐categories for this study were:

1. sweet biscuits, wafers, cookies;
2. savoury/salty snacks such as chips/crisps, crackers and *krupuk* (shrimp chips);
3. candy, chocolate and jellies/*agar‐agar*;
4. sweetened milks, defined as milks marketed for general consumption, with added sugar and flavour, excluding sweetened condensed milk; and
5. malt‐based beverages (e.g. Milo) and non‐dairy milks.

### 2.3 Data collection

Data were collected in 43 stores during May 2017. Grocery stores/supermarkets and hypermarkets were surveyed on the weekend as this was hypothesized to be the most likely time for promotions with company representatives. The baby stores and all small stores were surveyed on week‐days as previous scouting indicated that promotions appeared similar on weekends and week‐days.

To guide data collection on product availability, comprehensive master lists of all BMS and CPCF products for sale in Bandung City were generated through a review of the *Badan Pengawasan Obat dan Makanan* (National Food and Beverage Registry), online research and informal visits to seven stores. The final master lists included product brand, flavour, product description, age of use and manufacturer. Different flavours of a product were listed as different products. Products with varying package sizes (single‐serving verses multi‐serving, and different sizes of multi‐serving packages) and different types of packaging (e.g. box, sachet) were listed as one product. Each product was assigned a unique identification code for data collection and analysis. Master lists for the commercial snack products were not generated because the large number of products on the market made this logistically unfeasible.

In each store, data on product availability were captured first. All areas inside a store were thoroughly surveyed (e.g. baby food section, milk section, baby supplies section and discount section) to identify all products for sale. Enumerators systematically matched each BMS and CPCF product found throughout the store to a paper copy of the master lists, checking off each individual product identified for sale. Any products not found on the master lists were added to the list for all subsequent points‐of‐sale to be visited.

Once all information on product availability was recorded, enumerators captured information on each promotion observed throughout the store. A promotion was defined as an individual occurrence of promotional activity in a store for one or more BMS, CPCF or commercial snack product, such as a shelf display, price discount or an informational brochure (Champeny et al., 2016). The types of promotions assessed were:

1. price‐related, such as coupons, discounts or buy‐one‐get‐one;
2. displays, including brand shelves/counters/tables, special shop windows, posters/banners or shelf tags/talkers/wobblers;
3. information materials, like leaflets, pamphlets/brochures or catalogues;
4. free gifts to customers, such as toys, baby items or plastic food‐storage containers;
5. product samples;
6. company representatives in store;
7. store banners/signs with store name/logo and product logo/brand; and
8. any other promotions, such as contests, store bonus‐points or holiday baskets.

If a promotion included two distinct promotion types, each type was counted as a unique promotion. For example, a product placed in a special display booth with an offer of a free gift was counted as one display promotion and one free gift promotion.

For each promotion, enumerators recorded the type of promotion and type of product promoted (BMS, CPCF or snack). If BMS or CPCF were promoted, the unique product codes from the master lists were recorded for each product observed in the promotion. Promotions for BMS and CPCF without distinct product sub‐categories (i.e. a broad promotion for a brand/manufacturer, without indication of unique products that could be matched to the master list) were captured as “sub‐category cannot be identified.” For each snack product promoted, the snack sub‐category (e.g. sweet biscuit), brand and manufacturer were recorded. Promotion data were collected on electronic tablets using the Android application Open Data Kit (ODK), and uploaded to ONA, an open‐source online platform (ONA, 2018). Each promotion was photographed (WHO & UNICEF, 2017) and uploaded as part of the ODK survey.

### 2.4 Review of caregiver‐ or child‐targeted commercial snack promotions

After data collection was complete, photographs of the commercial snack promotions were reviewed for caregiver‐ or child‐targeting based on seven criteria adapted from previous research (Chacon, Letona, & Barnoya, 2013; Gantz, Schwartz, Angelini, & Rideout, 2007; McGinnis, Gootman, & Kraak, 2006; Mehta et al., 2012). Criteria for child‐targeted promotions included having (1) a gift for a child (e.g. toy, book and bottle) or (2) cartoons, licensed characters, images of children, sports images and/or animals on the promotion itself. Images appearing on the label of a package shown in the promotion were excluded. Criteria for caregiver‐targeted promotions included having (1) the word “baby,” “toddler,” “young child” or synonym; (2) the words “mom,” “dad” or synonym; (3) nutrient content claims (e.g. “contains iron” or “with DHA”), nutrient function claims (e.g. “promotes growth” or “improves intelligence”) or health claims (e.g. “enhances immunity” or “reduces indigestion”); (4) emotional cues (e.g. “happy” or “exciting”) or (5) statements directed towards caregivers about children (“we understand you only want the best for your child”).

Two analysts independently reviewed all snack promotions and their corresponding photographs in accordance with protocol used by Pereira et al. (2016) and Sweet et al. (2016). Promotions were graded “Yes” or “No” for each of the seven criteria. The two sets of grades were compared for agreement by a third analyst; 2.7% disagreement was found and a third analyst made the final determination.

### 2.5 Data analysis

Data on product availability were entered into Excel daily. Promotions data and photographs were reviewed by a study coordinator and then uploaded to ONA nightly. The grading of snack promotions was entered into Excel, and the check for agreement was run using Stata version 14 (StataCorp, College Park, TX, USA). Data were cleaned in Excel and all analyses were run in Stata.

The number of unique BMS and CPCF products observed across all stores was summed to calculate overall product availability. Availability was also disaggregated by sub‐category of BMS and CPCF, manufacturer and location of production (domestic or imported). The number of stores selling at least one type of product or one product sub‐category was calculated to report availability by store.

The number of stores with at least one promotion observed for each product type was determined. Percentage of stores with promotions was calculated by dividing the number of stores with promotions by the number of stores selling the product. Data on the availability of commercial snack products were not collected due to the overwhelming number of items available, so the percentage of stores selling snack products is reported out of all 43 stores in the study. Promotion data by store are also presented by small or large store size.

The number and percentage of available BMS and CPCF products that were found promoted across all stores were calculated by dividing the number of products promoted by the number of products found available for sale. If a product was documented in at least one promotion, it was counted as promoted. Promotion by availability was also calculated for each BMS and CPCF sub‐category.

The number and percentage of all promotions found across the 43 stores were calculated by type of product and sub‐category. If a promotion had at least one product type/sub‐category, it was included in the count of promotions. Promotions including multiple categories of products were classified as joint‐promotions, and the different combinations of product types were examined. The total numbers of BMS, CPCF and snack promotions were also disaggregated by type of promotion (e.g. price or display). Snack promotions were considered child‐targeted if they included at least one of the two criteria, and caregiver‐targeted if they included at least one of the five criteria.

## 3 RESULTS

### 3.1 Availability of breastmilk substitutes and commercially produced complementary foods

In the 43 stores surveyed, 147 unique BMS products were found: 44 infant formulas, 24 follow‐up formulas and 79 growing‐up milks. The BMS products were from 13 different manufacturers, with three producing over 60% of those observed: Nutricia (31.3%, *n* = 46), Nestle (15.6%, *n* = 23) and Kalbe (13.6%, *n* = 20). Sixty‐nine percent (*n* = 101) of all BMS products were domestically produced: 56.8% of infant formula products (*n* = 25), 70.8% of follow‐up formula products (*n* = 17) and 74.7% of growing‐up milk products (*n* = 59).

BMS products were available in 28 of the 43 stores surveyed (65.1%), in all 10 of the large stores and 18 of the 33 small stores (54.5%). The number of stores carrying the sub‐categories of BMS was similar, with 26 selling infant formula (60.5%), 25 selling follow‐up formula (58.1%) and 28 selling growing‐up milk (65.1%).

A total of 220 unique CPCF products were found. Infant cereal was the most common sub‐category of CPCF found (44.1%, *n* = 97), followed by snacks/finger foods (32.7%, *n* = 72), purees and infant meals (17.3%, *n* = 38), puddings (3.2%, *n* = 7) and other CPCF (2.7%, *n* = 6), which included thee olive oils, two teas and one *abon*. These items were produced by 20 different manufacturers, with three producing over half of all CPCF products: Indofood (21.8%, *n* = 48), Kraft Heinz (18.2%, *n* = 40) and Kalbe (14.1%, *n* = 31). Half of the 220 CPCF (53.6%, *n* = 118) were manufactured outside of Indonesia. The majority of infant cereal products (71.1%, *n* = 69) and all puddings (*n* = 7) were domestically produced, and most snacks/finger food products (69.4%, *n* = 50), purees and infant meals (92.1%, *n* = 35) and other CPCF products were imported (83.3%, *n* = 5).

CPCF were almost universally available across all 43 stores (97.7%, *n* = 42). Nearly all stores sold an infant cereal product (97.7%, n = 42) and half had snacks/finger foods (55.8%, *n* = 24). Purees and infant meals, puddings and other CPCF products were observed less frequently (20.9%, *n* = 9; 20.9% *n* = 9; and 9.3%, *n* = 4, respectively).

### 3.2 Promotions for breastmilk substitutes, commercially produced complementary foods and commercially produced snack products

BMS and CPCF products were promoted in the majority of the stores they were sold in (82.1% and 81.0%, respectively). Promotions were more prevalent for BMS and CPCF in large stores (90.0% and 100%, respectively) than small stores (77.8% and 75.0%). Almost half of stores selling BMS (42.9%, *n* = 12) had promotions for infant and/or follow‐up formula. Nearly all stores in the study (95.3%) promoted the study's subset of commercial snack products commonly fed to children.

**Figure 1:**
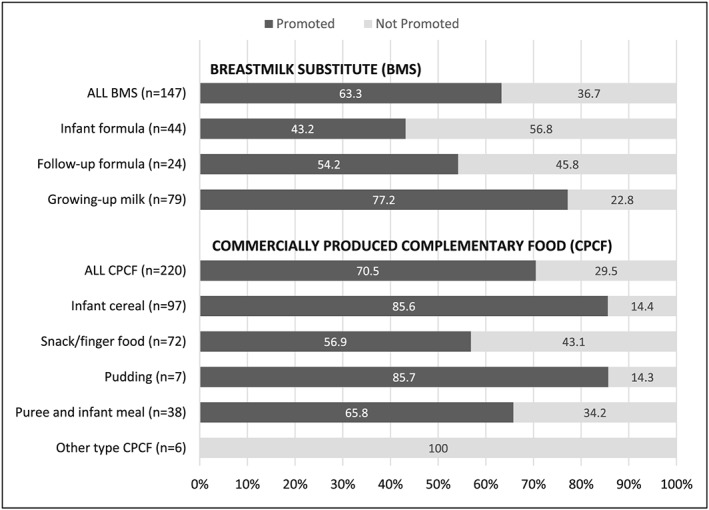
Percentage of available breastmilk substitute and commercially produced complementary food products that were found promoted in stores. Number of products found available for sale reported as (*n*)

Figure 1 shows the percentage of infant and young child food products available for sale that had at least one promotion. Almost two‐thirds of the 147 unique BMS products were promoted, with more of the growing‐up milks promoted (77.2%) than infant formula (43.2%) and follow‐up formula (54.2%). Seventy percent of CPCF products were promoted, including 85% of the available infant cereal and pudding products.

Across all stores, a total of 402 promotions included at least one BMS product, 206 included at least one CPCF product and 2,451 included at least one commercially produced snack product (Table 1). Nearly all BMS promotions included a growing‐up milk and 16 promotions included infant formula and/or follow‐up formula (4.0% of all BMS promotions). Infant cereal was the most commonly promoted sub‐category of CPCF. All sub‐categories of snacks were similarly promoted except for malt‐beverages/non‐dairy milks.

**Table 1:** Number and percentage of promotions for breastmilk substitutes, commercially produced complementary foods and commercially produced snack products observed in points‐of‐sale, by product sub‐category^a^

Product category and sub‐category	All promotions observed	
	*n*	%
Breastmilk substitute (BMS)		
Infant formula	15	3.7
Follow‐up formula	13	3.2
Growing‐up milk	395	98.3
Sub‐category undetermined^b^	8	2.0
Any BMS product	402	100
Commercially produced complementary food (CPCF)		
Infant cereal	151	73.3
Snack/finger food	47	22.8
Pudding	14	6.8
Puree and infant meal	7	3.4
Other type product	0	0
Sub‐category undetermined^b^	7	3.4
Any CPCF product	206	100
Commercially produced snack product		
Sweet biscuit	782	31.9
Savoury snack	683	27.9
Candy, chocolate, jellies	588	24.0
Sweetened milk	704	28.7
Malt‐beverage, non‐dairy milk	105	4.3
Any commercial snack product	2451	100

a Possible for a promotion to include more than one product category or sub‐category.

b Sub‐category of product could not be determined in the promotion.

Promotions were found to promote one category of product or multiple categories of products together, such as BMS and CPCF (Table 2). Examples of joint‐promotions included displays or posters showing different products together, company representatives promoting a range of products or buy‐one get‐one half‐price discounts. BMS products were more frequently included in joint‐promotions (197) than CPCF (21). All the BMS joint‐promotions included commercial snack products (197) and comprised 49.0% of all BMS promotions. Of these joint‐promotions, 190 included BMS and a sweetened milk (47.3% of all BMS promotions) and 183 included a growing‐up milk and a sweetened milk (45.5% of all BMS promotions). Table 3 shows the sub‐categories of BMS and commercial snack products in the 197 joint‐promotions. Eighty percent of all infant formula promotions and 84.6% of all follow‐up formula promotions included a commercial snack.

**Table 2:** Number and percentage of all promotions observed by category of product included in the promotion

Category of product included in the promotion	All promotions observed (*n* = 2,841)	
	*n*	%
Only breastmilk substitute (BMS)	205	7.2
Only commercially produced complementary food (CPCF)	185	6.5
Only commercially produced snack (snack)	2241	78.9
BMS + CPCF	0	0
BMS + snack	189	6.7
CPCF + snack	13	0.5
BMS + CPCF + snack	8	0.3

**Table 3:** Percentage and number of joint‐promotions of breastmilk substitutes and commercially produced snack products, by sub‐category of breastmilk substitute and snack product^a^^,^ b

Sub‐category of commercial snack	Products in joint‐promotion		
	Infant formula and commercial snack (*n* = 12)	Follow‐up formula and commercial snack (*n* = 11)	Growing‐up milk and commercial snack (*n* = 197)
Sweet biscuit	33.3 (4)	36.4 (4)	12.2 (24)
Savoury snack	33.3 (4)	36.4 (4)	12.2 (24)
Candy, chocolate, jellies	41.7 (5)	45.5 (5)	10.2 (20)
Sweetened milk	100 (12)	100 (11)	96.2 (190)
Malt‐beverage, non‐dairy milk	25.0 (3)	27.3 (3)	9.6 (19)

a Data presented as percentage (*n*).

b Possible for a promotion to include more than one sub‐category of snack.

BMS products were jointly promoted with CPCF products in eight promotions (Table 2), although only in conjunction with a commercial snack product. CPCF products were jointly‐promoted with commercial snacks in 21 promotions; 10.2% of all CPCF promotions observed. Of these 21 joint‐promotions, 90.5% (*n* = 19) included sweetened milk, followed by 71.4% (*n* = 15) with savoury snacks, 61.9% (*n* = 13) with malt‐beverages/non‐dairy milks, 57.1% (*n* = 12) with sweet biscuits and 38.1% (*n* = 8) with candy/chocolate/jellies.

**Figure 2:**
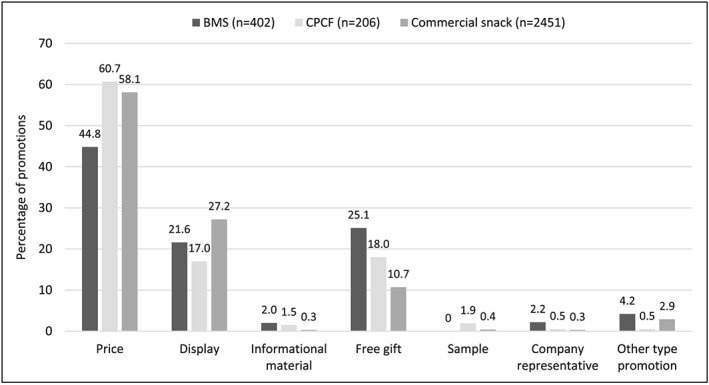
Percentage of promotions by type of promotion and product type. BMS: breastmilk substitute; CPCF: commercially produced complementary food. Due to rounding, percentages may not add up to 100%. Data not shown for store banner/sign: 0% BMS, 0% CPCF and <0.1% commercial snack

Price‐related promotions were the most common method of promotion, accounting for 44.8% of all BMS promotions, 60.7% of CPCF promotions and 58.1% of commercial snack promotions (Figure 2). Display promotions were also prevalent across all three types of product as well as free gift promotions.

Table 4 presents the types of promotions for the sub‐categories of BMS. Promotions of infant formula and follow‐up formula were mostly display promotions, and the majority of promotions with growing‐up milks were price promotions. Though only a small proportion of all BMS promotions, company representatives were observed promoting growing‐up milks or BMS brands/manufacturers in general.

**Table 4:** Percentage and number of promotions by type of promotion and breastmilk substitute sub‐category^a^^,^ b

	Sub‐category of breastmilk substitute			
Type of promotion	Infant formula (*n* = 15)	Follow‐up formula (*n* = 13)	Growing‐up milk (*n* = 395)	Sub‐category undetermined^c^ (*n* = 8)
Price	20.0 (3)	15.4 (2)	45.3 (179)	0 (0)
Display	53.3 (8)	53.8 (7)	22.0 (87)	25.0 (2)
Informational material	0 (0)	0 (0)	2.0 (8)	0 (0)
Free gift	20.0 (3)	23.1 (3)	25.3 (100)	0 (0)
Company representative	0 (0)	0 (0)	1.0 (4)	75.0 (6)
Other type of promotion	6.7 (1)	7.7 (1)	4.3 (17)	0 (0)

a Data presented as percentage (n). Due to rounding, percentages may not add up to 100%.

b Possible for a promotion to include more than one sub‐category of breastmilk substitute.

c Sub‐category of product could not be determined in the promotion.

Although overall the most common type of CPCF promotion was price promotion, the type of promotion varied by sub‐category of CPCF. Price promotions continued to be common for promotions with snacks/finger foods (68.1%, *n* = 32), infant cereal (57.0%, *n* = 87) and puddings (42.9%, *n* = 6). Gift promotions were highly prevalent for purees and infant meals (57.1%, *n* = 4) and puddings (42.9%, n = 6). Price promotion was also the most frequently observed promotion method for all commercial snack product sub‐categories, followed by display promotions.

### 3.3 Child‐ and caregiver‐targeting of commercial snack promotions

**Figure 3:**
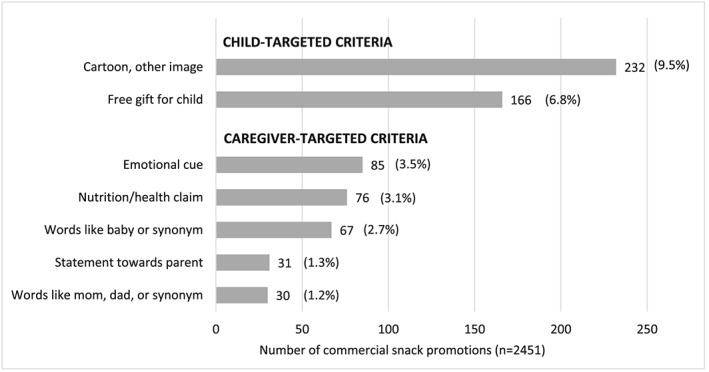
Number and percentage of all commercially produced snack promotions by type of child or caregiver targeting criteria. Data presented as n (percentage)

In the review of 2,451 promotions with commercial snack products, 17.3% (*n* = 425) met at least one of the criterion for child‐ or caregiver‐targeting. In total, 214 (8.7%) were child‐targeted, 119 (4.9%) were caregiver‐targeted and 92 (3.8%) were both child‐ and caregiver‐targeted. Figure 3 shows the number and percentage of all snack promotions meeting each criterion. Promotions of candies, sweet biscuits and savoury snacks were targeted more to children (24.8%, 11.1% and 7.2%, respectively) than caregivers (6.1%, 5.4% and 3.1%). Sweetened milks and malted beverages/non‐dairy milks were similarly targeted to children and caregivers: sweetened milks, 17.1% child‐targeted and 22.3% caregiver‐targeted, and malted beverages/non‐dairy milks, 22.9% child‐targeted and 18.1% caregiver‐targeted.

## 4 DISCUSSION

Promotions for BMS, CPCF and commercial snacks commonly fed to young children were highly prevalent in this study of 43 stores in Bandung City, Indonesia. Over 80% of stores carrying BMS and CPCF products had promotions for these products, and commercial snack promotions were nearly universal. Nearly two‐thirds of all BMS products and three‐quarters of CPCF products available for sale were being promoted. The majority of BMS promotions were for growing‐up milks for children older than 1 year; however, we found promotions for infant and follow‐up formula, which violates national regulations. Manufacturers frequently promoted BMS jointly with commercial snack products, mainly sweetened milks. Nearly 2,500 promotions were observed for five categories of commercial snack products, with almost one‐fifth targeting children and/or their caregivers.

BMS promotions that break both international standards and national laws were identified in our research. Promotion of any BMS category (infant formula, follow‐up formula or growing‐up milk) violates the Code and subsequent resolutions. In addition, Indonesian law prohibits promotion of BMS products for children under 12 months of age. Although only 4.0% of all BMS promotions were in violation of national regulations, these were not isolated promotions. They were observed in 42.8% of all stores selling BMS and promoted half of all the infant and follow‐up formula products for sale. Moreover, these products were frequently found in joint‐promotion with commercial snack products.

The promotion of growing‐up milks was rampant—98.3% of all BMS promotions, showcasing three‐quarters of all growing‐up milk products available for sale—and strongly reinforces the need to expand the scope of age covered in national regulations. Recent research in Bandung City found that 47.0% of children 12–23 months and 55.1% of children 24–35 months consumed a BMS in the prior day (HKI & MOH, 2018a). WHO recommends against the use of growing‐up milks and notes that current formulations lead to higher protein intake and lower intake of essential fatty acids, iron, zinc and B vitamins than those recommended for adequate growth and development of infants and young children (WHO, 2013). Continued breastfeeding in the second year of life protects against mortality and makes important and unique contributions to the dietary intake of young children (Sankar et al., 2015). Although the WHO recommends breastfeeding for up to 2 years or beyond, most legislation against the promotion of BMS, including Indonesia's, applies only to products through the first year of life (WHO, UNICEF, & IBFAN, 2016). Several countries with strict regulations against the promotion of BMS have seen increased rates of breastfeeding (Brady, 2012; Bragg, Hardoby, Pandit, Raji, & Ogedegbe, 2017; Champeny et al., 2016; Cyrillo, Sarti, Farina, & Mazzon, 2009). In countries like Indonesia where promotion for and use of growing‐up milks is rising, there is a pressing need to expand restrictions against the promotion of BMS for children up to three years of age.

The pervasive promotions for growing‐up milks found by this study is also troubling because previous research has shown that mothers and caregivers often cannot differentiate between the different stages of BMS products (Berry, Jones, & Iverson, 2010; Cattaneo et al., 2015) and caregivers confused by similar packaging could inappropriately use growing‐up milks to feed younger infants. Pereira et al. (2016) documented BMS manufacturers using the same brand attributes across their range of products in four study sites. The majority of follow‐up formulas and growing‐up milks manufactured by infant formula companies displayed at least one example of cross promotion with one or more of the company's infant formula products: two‐thirds or more contained similar colour schemes, designs and mascots. Furthermore, growing‐up milk advertisements function as indirect advertising for infant and follow‐up formula (Berry et al., 2010; Cattaneo et al., 2015). In the absence of regulation prohibiting cross promotion through similar packaging and labelling, the widespread retail promotion of growing‐up milks in Bandung City could serve to indirectly promote a manufacturer's entire range of BMS, circumventing restrictions on BMS promotion and undermining breastfeeding.

CPCF products were found in nearly every store surveyed, indicating the ubiquity of commercially available complementary foods. Over 200 promotions were observed for 70.5% of the CPCF available for sale, which is not necessarily negative as it has long been recognized that suitable CPCF can play a role in maintaining appropriate nutritional intake for young children, especially when foods are fortified (Brown & Lutter, 2000; Dewey, 2009). A 2011 study of 12‐ to 24‐month‐old children in Bandung City found that, “fortified food made a large contribution to nutrient adequacy for many micronutrients in the children's diet. This suggests that if fortified foods are omitted from the children's diet, the proportion of children who meet nutrient recommendations will dramatically drop” (Santika & Fahmida, 2015). WHO guidance asserts that fortified complementary foods can play an important role in child feeding (PAHO & WHO, 2003). The WHA Resolution 69.9 states that, “foods for infants and young children that are not products that function as breast‐milk substitutes should be promoted only if they meet all the relevant national, regional and global standards for composition, safety, quality and nutrient levels and are in line with national dietary guidelines” (WHO, 2016). This guidance presumes that products are fortified with both the appropriate amounts and types of nutrients, which is an area of investigation and legislation where the Indonesian government needs to engage in order to fulfil the WHA guidance (Dreyfuss et al., 2019).

Joint‐promotions in which products were directly marketed together were also widely observed. BMS products were commonly promoted with other BMS products and commercial snack products. Sweetened milks in particular were commonly used in joint‐promotions, appearing with infant formula, follow‐up formula, growing‐up milks and CPCF products. Many of these sweetened milks contain 12–19 g of added sugar per single serving package (200–250 ml; Green, unpublished observation), promoting excess sugar consumption. A recent multi‐country study documented other instances of placement of BMS and CPCF in the same promotion at points‐of‐sale (Champeny et al., 2016). This kind of joint‐promotion could lead to early introduction of complementary foods, confusion about age and product categories, and what constitutes appropriate, nutritious foods for children (Champeny et al., 2016; Smith et al., 2015; Sweet et al., 2016), and needs to be regulated.

This study is one of the first to assess targeting of commercial snack products to children under 3 years and their caregivers in Indonesia, and the prevalence of targeting found is concerning. Promotions of commercially produced snack foods and sugar sweetened beverages have been shown to influence purchase and consumption behaviours (Boyland et al., 2016; Sonntag, Schneider, Mdege, Ali, & Schmidt, 2015). In our study, promotions for candy/chocolate/jellies and malted beverages/non‐dairy milks, which are often high in added sugar, were found to most frequently use techniques that targeted children. The most prevalent caregiver‐targeted promotion was for sweetened milks (22.3%), which is also troubling as a recent multi‐country study on the perception of beverage healthiness found that Indonesian participants perceived flavoured, sweetened milk to be healthier than other sugar‐sweetened beverages (Thomson et al., 2017). With prevalent consumption of unhealthy commercial snack products by young children in Indonesia (Green et al., 2019), and rising rates of child overweight/obesity (MOH, 2013; Rachmi, Li, & Baur, 2017), promotions for these products could have a significant impact on the health and well‐being of children in Indonesia. The Indonesian government should consider passing and enforcing food industry regulations related to caregiver‐ and child‐targeted promotion of commercial snack foods to reduce the impact of unhealthy snacking among children.

This study has several limitations. Stores sampled for this study were not representative of all vendors in the city; however, WHO's recommended NetCode protocol was followed and 44.2% of the stores (*n* = 19) were local or national chain stores, so promotions observed were likely to be found throughout the city and country. For commercial snack products, only a subset of categories was examined, which does not represent the totality of snack promotions or totality of promotions for commercial foods high in fat, sugar or salt in these stores. Additionally, we did not assess purchase or consumption of products so it is not known if promotion in this research led to greater consumption of these products. This assessment was carried out with assumptions about store characteristics and retail behaviour, which could have introduced bias: different store types were visited at different times/days of the week, which could have led to differentials in promotion observation, and the store sampling methodology differed between small and large store types.

## 5 CONCLUSION

This research conducted in large and small stores across Bandung City, West Java, Indonesia, has revealed numerous promotions for BMS, CPCF and commercially produced snack products, with several BMS promotions illegal under Indonesia law. A high prevalence of child‐ and caregiver‐targeted snack promotions was found, along with frequent joint‐promotion of BMS, CPCF and commercial snacks. Overall, these promotions may be contributing to decreased breastmilk intake and/or increased consumption of trans‐fats, sugar and salt among young children, ultimately leading to long‐term health consequences. A national system for monitoring and reporting violations of the *International Code of Marketing of Breast‐Milk Substitutes* and subsequent WHA resolutions, with significant repercussions for violators, would be useful in curbing industry violations. Expansion of current national laws to align with WHO guidance and protection of young children from the harmful effects of unhealthy consumption is urgently needed.

## CONFLICTS OF INTEREST

The authors declare that they have no conflicts of interest.

## CONTRIBUTIONS

DH, MG, AS and MC prepared the manuscript, with input from DI. DH, A and MG collected the data, A coded data and MG oversaw data collection and coding. MG cleaned and analysed the data. AS guided data interpretation and provided technical insight. All authors reviewed and provided input on the final article.
